# Reasons for Placement and Replacement of Resin-based Composite Restorations in Greece

**DOI:** 10.5681/joddd.2011.020

**Published:** 2011-09-05

**Authors:** Nikolaos Andreas Chrysanthakopoulos

**Affiliations:** ^1^Dental Surgeon (DDS), Resident in Maxillofacial and Oral Surgery, 401 General Military, Hospital of Athens, Athens, Greece

**Keywords:** Dental caries, longevity, replacement, resin composite restorations

## Abstract

**Background and aims:**

The purpose of this investigation was to assess the placement and replacement of resin-based composite restorations and related factors in a private practice in Greece.

**Materials and methods:**

The study included 1500 subjects, 720 males and 780 females, aged 18 to 52 years old. The clinical examination involved calculation of the number of teeth with primary caries and failed-restored teeth. In addition the relationship between placed and replaced composite restorations and the following aspects was assessed: gender, cavity type, tooth type and evaluation of longevity of the replaced composite restorations. Statistical analysis performed using the chi-square test. A p value less than 5% was considered statistically significant.

**Results:**

The total number of restorations placed were 1940; 1202 of those (62%) were placed for first time while 738 (38%) were replaced. The main reason for the placement of new composite resin restorations was primary caries (60%), while secondary caries was the most frequent reason for the replacement (48%) of those. A statistically significant differ-ence was recorded between males and females regarding the composite restorations placed and replaced (p = 0.00082), the type of cavity of placed restorations (p = 0.00062), and the type of cavity of replaced ones (p = 0.00038). The median lon-gevity of the replaced resin composite restorations was approximately 4 years (47%).

**Conclusion:**

Dental caries, primary and secondary, followed by tooth discoloration and loss of filling were the main rea-sons for placed and replaced composite restorations.

## Introduction

First resin-based composite material was introduced as a Class II restorative in 1968, but soon failed because of several reasons such as tooth sensitivity, recurrent caries, open contact areas, fracture of the restorative material, excessive wear and discolored surface.^1^ Longevity and failure of restorations, in general, could be attributed to several factors such as the clinical usage of the material, patient’s compliance and clinician’s decision regarding the indications for placement and replacement of restorations.^2^

Resin-based composite materials are considered as the materials of choice for conservative esthetic restorations of mainly Class I–V cavities or traumatic injuries. Previous studies implicated primary caries as principal reason for placement of restoration,^3 - 12^ while secondary caries was the most frequent reason for replacement of composite restorations followed by tooth discoloration and marginal fracture. ^5, 7 - 20^, Several reasons have been identified regarding the replacement of composite restorations such as fracture and marginal defect of the restoration, tooth fracture, marginal staining of tooth, deficient anatomical form, and over-contouring of the restorations. ^7, 8, 11, 13, 15, 17, 19 - 21^

Mjor^22^ reported that secondary caries and poor marginal adaptation were the most common reasons for failure of composite resin restorations and mentioned that failure of restorations was a major problem in dental practice, as replacements comprise about 60% of all operative work. Another study by Brukiene et al.^23^ showed that, in many cases, the failure of a composite resin restoration does not only depend on the material itself but also on the proper handling of it.

In general, the reasons for the replacement vary depending on the restorative material, the dentition, and the age of the patient.^28 , 29^ The aim of the present study was to assess the reasons for placement and replacement of resin-based composite restorations and to evaluate the relationship between placed and replaced composite restorations by gender, type of cavity, tooth type and longevity of replaced restorations.

## Materials and Methods

###  Subjects

Study population consisted of 1500 subjects, 720 males and 780 females, aged 18-52 years (mean age 38.3 ± 6.5 years) who sought dental treatment in a private practice in Patra, one of the biggest cities in Greece.

The reasons for placement and replacement of composite restorations of the sample for a period of two years (October 2008-November 2010) were obtained including aspects such as gender, type of cavity (according to Black’s classification), location/type of restored teeth and longevity of replaced restorations, according to self-reported questionnaires regarding the age of their failed composite restorations. It is therefore not possible to calculate objectively the longevity of all replaced restorations. A comprehensive history was taken and all examinations were performed by the author in private practice. The participants were in good general health as estimated by a health questionnaire.

### Ethical considerations

The present study was not an experimental one. In Greece, only experimental studies must be reviewed and approved by authorized committees (Dental Schools, Greek Dental Associations, Ministry of Health, etc.). Subjects who agreed to participate in the present study were informed about the evaluation to which they would be submitted and signed an informed consent form.

Patients with diagnosed pathological conditions were advised to seek consultation and treatment.

### Clinical Examination

The clinical measurements of the participants were performed by the author. The teeth and gingiva were dried with compressed air while dental unit light was used as the light source for the inspections. Restored and non-restored teeth were examined carefully using an intra-oral mirror and an explorer.

The main criteria which indicated the placement of restorations were those determined by the W.H.O,^24^ and focused on the clinical signs of primary caries and presence of carious lesion, namely lesions of grooves, vents, crevices, and smooth surfaces which had soft substrates, those that appeared as grey areas and those that developed visible cavity. The main criteria which indicated the replacement of restorations were those determined by the California Dental Association Quality Evaluation System^25^ and included the following for the assessment of the quality of dental restorations:

- surface quality and color,
- anatomical form,
- margin integrity.

### Inclusion and exclusion criteria

The selection criteria comprised age above 18 years and a minimum number of 20 natural teeth, since large numbers of missing teeth, i.e. more than 12 missing teeth, can cause problems with eating, speech, and other basic activities that may worsen with time. Eventually, the remaining teeth in the jaw shift in an attempt to fill in the gap left by a missing tooth. That situation can cause other oral diseases, including periodontal disease (pathologic migration, mobility) temporo-mandibular joint (TMJ) disorder, and dental caries.^26^

Only anterior teeth of the mandible and maxilla were included in the present study. Restored and non-restored molars and premolars and amalgam-restored anterior teeth were excluded from the study.

### Restorations

A resin-based composite material (Filtek Z250, 3M ESPE, St. Paul, U.S.A.) was used for the placement and replacement of restorations of the anterior teeth. The polymerization of the material performed by using of a Dental Light Cure Unit Halogen Type (LK-G21) (technical parameter: output power 75 W, wave length 400-500 nm, light power 800-1000 mw/cm^2^, solidification time and depth 20s/2mm). The material used is ideal for restorations of anterior teeth and unsuitable for restorations of posterior teeth because of its mechanical properties, the reason why posterior teeth were excluded from the study. The bonding and restoration process was the following, in general, for placement and replacement of composite restorations: Preparation of tooth and isolation; application etchant (Scotchbond, 3M ESPE, St. Paul, U.S.A.) to enamel and dentin for 15 seconds and rinse; application of adhesive (Single Bond, 3M ESPE, St. Paul, U.S.A.) to enamel and dentine using a brush tip; drying gently for 2-5 seconds and light curing for 20 seconds;- placement of restorative material in increments less than 2.5 mm and light curing each increment for 20 seconds; and finishing and polishing.

### Statistical analysis

Statistical units of the present study were the individual and the tooth. For each subject the number of decayed (primary caries) and failed-restored teeth was calculated. Chi-square test was employed to test the hypothesis of no differences between males and females regarding the number of placed and replaced restorations (Class I-III), the reasons for placement and replacement of the restorations, the type of tooth with placed and replaced restorations, and the longevity of restorations. The data analysis was performed using the SPSS 16.0 software package (SPSS Inc., Chicago, IL). A p value less than 5% (p<0.05) was considered as statistical significance level.

## Results

A total of 1940 composite resin restorations were placed during the present study, 931 (48%) in males and 1,009 (52%) in females; 1,202 of those (62%) were placed for first time while 738 (38%) were replaced.

More than half of the placed composite restorations were made for males (54%) and 46% for females, while 28% of the replaced composite restorations were made for males and 72% for females. The difference between males and females regarding the composite restorations placed and replaced was statistically significant (p = 0.00082).

The distributions of composite resin restoration according to Black’s Class V, Class IV, and Class III were 35%, 17%, and 48%, for first-time placement and 12%, 36%, and 52%, for replacement, respectively. A statistically significant difference was recorded (p = 0.0044) between placed and replaced composite restorations and the type of cavity according to Black’s classification.

**Figure 1:**
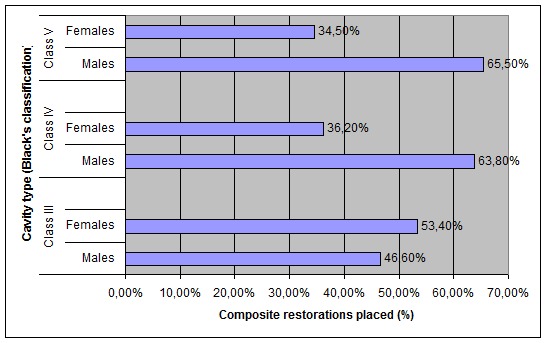
Distribution of placed composite restorations according to Black’s classification by gender.

Males showed a higher frequency of composite restorations placed than females regarding Class IV and V; the difference between males and females regarding the type of cavity of placed restorations was statistically significant (p = 0.00062) (Figure 1).

**Figure 2:**
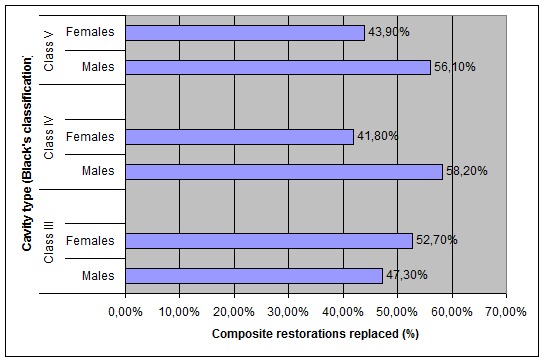
Distribution of replaced composite restorations according to Black’s classification by gender.

**Figure 3:**
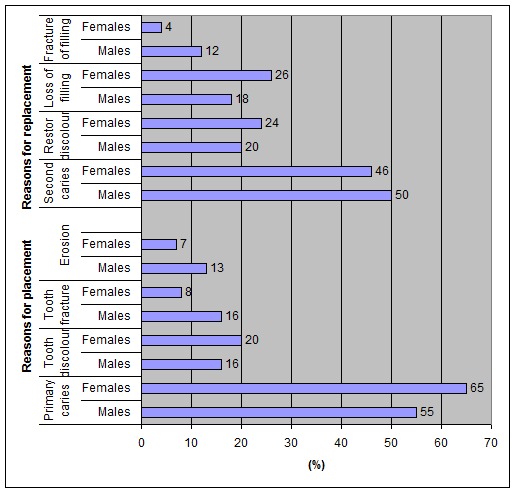
The reasons for placement and replacement of composite restorations by gender.

**Figure 4:**
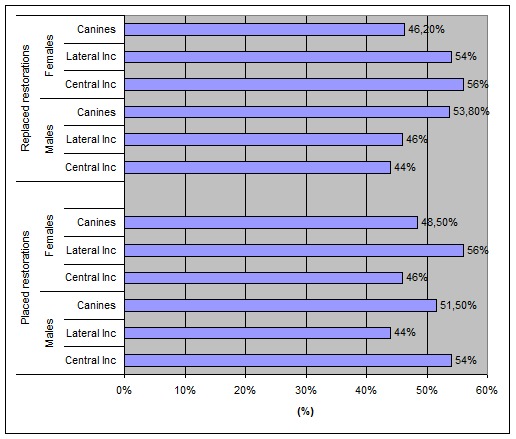
Distribution of placement and replacement composite restorations according to tooth type by gender.

The same observations were recorded for the replacement of composite restorations between males and females (p = 0.00038) (Figure 2). Primary caries was the principal reason for placement of composite restorations for both genders (60%) followed by tooth discoloration (18%), tooth fracture (12%) and erosion (10%); however, the difference between males and females was not statistically significant (p = 0.563; Figure 3). Secondary caries (48%) was the principal reason for replacement of composite resin restorations for both genders followed by restoration discoloration (22%), loss of filling (22%) and filling fracture (8%) with no statistically significant difference between males and females (p = 0.852; Figure 3). The distribution of replacement and placement restorations according to tooth type for both genders is shown in Figure 4. There were no statistically significant differences between males and females regarding the frequencies of placement of restorations (p = 0.788) and the replacement restorations (p = 0.429) according to tooth type.

The longevity of replaced composite restorations was recorded for 738 defective restorations. The median longevity of a composite restoration was approximately 4 years (47%), 20% of composite restorations were replaced in a period more than 8 years, 12% of those were replaced between 4-8 years and 21% were replaced in a period less than 1 year.

The median longevity of replaced composite restorations was <1 year in 18%, 1-4 years in 50%, 4-8 years in 8%, and >8 years in 24% of male subjects, and <1 year in 24%; 1-4 years in 44%; 4-8 years in 16% and >8 years in 16% of females evaluated. The difference between males and females regarding the longevity of replaced composite restorations was statistically significant (p = 0.00065).

## Discussion

Data collected in the present study show that primary caries was the principal reason for placing composite resin restorations. Similar results were observed in previous studies. ^3- 9, 11^ Braga et al.^10^ have shown that the main reasons for the placement of initial restorations were primary caries and non-carious tooth substance loss, while Frost^12^ found it to be, among others, repairs to tooth fractures. In this study, the main reasons for replacement of the restorations were secondary caries and discoloration, findings that are in accordance with those of previous studies. ^5, 7- 9, 13 - 17^ Previous research has shown that secondary caries is the principal reason for replacement of restorations.^10 , 12^ The high incidence of secondary caries associated with the composite restorations could be explained on the basis of microbiological findings.^27^ It is important to emphasize that composite restorations are extremely technique sensitive. Additionally, the ultimate clinical outcome is highly influenced by the oral hygiene of the patients. Composite accelerates the growth of Streptococcus mutans, which in combination with poor oral hygiene may cause secondary caries.^28^

A significantly higher proportion of Streptococcus mutans was found at the cavity margins of the composite restorations compared with those of amalgam and glass-ionomer material.^4^ In addition, another study by Friedl et al.^29^ showed that more dental plaque was found at the composite/tooth interfaces than at the amalgam/tooth interfaces, while observations from previous studies indicated that resin-based materials accumulate more dental plaque and the composition of this plaque is more cariogenic than that seen on amalgam, silicate cement and glass-ionomer materials.^27 , 29^

Another factor that has been associated with the development of secondary caries is microleakage.^30^ Pimenta et al.^31^ showed that crevices at the tooth-restoration interface of less than 35 to 50 μm do not predispose a patient to the development of secondary caries while larger crevices do. However, the bulk of available evidence indicates that there is no relationship between the development of secondary caries and the size of the crevice at the tooth-restoration interface except in cases of macro-leakage in which the crevice exceeded 250 or 400 μm. ^31, 32^ Thus, secondary caries do not develop as a result of micro-leakage along the tooth-restoration interface but it is a surface lesion similar to primary carious lesion on smooth surface.^33^

Another factor which leads to secondary caries is that all composites shrink during curing period, and thus it is important to minimize the effect of composite shrinkage following the usage instructions of the materials.^34^

Discoloration as a reason for replacement of composite restorations still remains a significant problem both for the clinician and the patient. A study by Mjor and Toffenetti^15^reported that margin discoloration suggests inadequate acid-etching of the enamel prior to placing the bond agent, inadequate handling of the material (placing, concentration, adaptation) and problems associated with polymerization shrinkage.

The increase in etched surface area results in a stronger enamel to resin bond, which increases the retention of the restoration and reduces marginal micro-leakage and marginal discoloration.^35^

Tyas^16^ showed that the main reasons for replacement of composite restorations were marginal discoloration, marginal fracture, and degradation. Those findings agree broadly with studies carried out from the United Kingdom.^6 , 7^

Previous studies indicated that tooth fracture was an additional reason for replacement of restorations, ^7, 11, 13, 15, 17, 19, 20^ while Vehkalahti and Palotie^36^ showed that secondary caries, along with fractures, overhangs and marginal discrepancy was the most common reason for replacement of restorations. Mjor and Gordan^21^ have identified the following reasons for replacement of resin restorations: restoration fractures, marginal infiltration, deficient anatomical form and over-contouring of the restorations, while Al-Negrish^8^ recorded the root canal treatment as the third important reason for replacement of restorations. It is important to notice that the reasons for the replacements vary depending on the restorative material; in many cases, the failure of a restoration does not only depend on the material itself, but also on proper handling of the material, the dentition and the age of the patient.^23^ The above differences could be attributed to the heterogeneous population samples examined, the progression of dental caries and the restorative materials during the past decades, the different methods and criteria used in order to assess the frequency of placed and replaced restorations (e.g. in some studies the investigators assessed placed and replaced composite restorations in anterior teeth or posterior teeth while in other studies amalgam restorations have been included), the different attitudes of the population samples regarding the value of teeth maintenance, and the need for a regular dental follow-up. The present study concerned subjects who sought dental treatment in a private practice and, therefore, the sample could not be considered as a random one.

According to the results of the present study the most placed composite restorations were rendered in male patients, while the most replaced composite restorations were done for females. Gender differences regarding the placement of new restorations could be attributed to the fact that females visit their dentists more frequently than males, while the differences regarding the replacement of composite restorations could be attributed to factors concerning the restorative material, dentition, and the age of the patient. In studies conducted in Norway and Iceland, no association was found in the reasons for replacement of restorations and patient gender,^5 , 13^while significant associations between gender and the reasons for replacement of restorations were recorded in a study by Asghar et al.^17^

The decision to replace a restoration is influenced by more subjective factors such as dentists’ interpretation of the restoration’s condition and the health of the tooth, the criteria used to define failure, and patient demand. These decisions are subject to a great deal of variation. There is a lack of standardization and no generally agreed criteria are used to decide when a restoration requires replacement.^37^

The median longevity of the failed restorations of the present study was approximately 4 years, while several studies have shown different results. A study by Mjör and Toffenetti^15^ showed that the median longevity was 3.3 years, while others reported the median longevity of composite restorations to be 3 years or 6 years.^17 , 18^ Similar studies reported survival periods of 7.1 years,^16^ 7.8 years,^19^ 6 years,^20^ 8 years,^38^ and 9 years.^39^

Vehkalahti and Palotie^36^showed that the mean age of failed restorations was 2.4 years while another study by Moorhead et al.^40^showed that the median age of resin-based composite restorations was 8 years.

Mjor et al^38^ showed that cavity form, preparation and careful handling of the material are prerequisites for longevity of restoration while the longevity period was influenced by the type and size of the restoration, the material and possibly the intra-oral location of the restorations.

It is difficult to discover specific reasons for the low median longevity of the restorations replaced; however, operative technique, material quality, and careful handling according to producer instructions may play important roles.^40^

As mentioned above, the success or failure of restoration, placed and replaced, depends on the following main factors: the dentist’s skills, the patient compliance and the restorative material, factors that are closely related. In addition, the oral hygiene of the patient may also play an important role in the development of secondary caries and discoloration.

## Conclusion

1. The principal reason for the placement of composite restorations was primary caries followed by tooth discoloration, tooth fracture and erosion for both genders, while the principal reason for the replacement of composite restorations was secondary caries followed by tooth discoloration, loss of filling, and filling fracture for both genders.
2. Most placed and most replaced restorations were Class III.
3. In males, the most placed restorations concerned the central incisors of both jaws, while in females they concerned the lateral incisors. The most replaced restorations in males concerned the canines of both jaws, while in females they were the central incisors of both jaws.
4. Most placed composite restorations were made for males while, most replaced ones were made for females.
5. The median longevity of a composite restoration was approximately 4 years (47%), 20% of composite restorations were replaced in a period more than 8 years, 12% of those were replaced between 4-8 years while 21% were replaced in a period less than 1 year.
